# Crystal structure and Hirshfeld surface analysis of (*E*)-3-[(2,3-di­chloro­benzyl­idene)amino]-5-phenyl­thia­zolidin-2-iminium bromide

**DOI:** 10.1107/S2056989018010496

**Published:** 2018-07-27

**Authors:** Mehmet Akkurt, Gulnara Sh. Duruskari, Flavien A. A. Toze, Ali N. Khalilov, Afat T. Huseynova

**Affiliations:** a Department of Physics, Faculty of Sciences, Erciyes University, 38039 Kayseri, Turkey; b Organic Chemistry Department, Baku State University, Z. Xalilov str. 23, Az, 1148 Baku, Azerbaijan; c Department of Chemistry, Faculty of Sciences, University of Douala, PO Box 24157, Douala, Republic of Cameroon

**Keywords:** crystal structure, iminium salt, thia­zolidine ring, 2,3-di­chloro­benzene, hydrogen bonding, Hirshfeld surface analysis

## Abstract

In the cation of the title salt, C_16_H_14_Cl_2_N_3_S^+^·Br^−^, the central thia­zolidine ring adopts an envelope conformation. The phenyl ring is disordered over two sites with a refined occupancy ratio of 0.541 (9):0.459 (9). In the crystal, C—H⋯Br and N—H⋯Br hydrogen bonds link the components into a three-dimensional network with the cations and anions stacked along the *b-*axis direction. Weak C—H⋯π inter­actions, which only involve the minor disorder component of the ring, also contribute to the mol­ecular packing. In addition, there are also inversion-related Cl⋯Cl halogen bonds and C—Cl⋯π (ring) contacts. A Hirshfeld surface analysis was conducted to verify the contributions of the different inter­molecular inter­actions.

## Abstract 2

In the crystal, the cations and anions stacked along the *b-*axis direction are linked by C—H⋯Br and N—H⋯Br hydrogen bonds, forming a three-dimensional network. In addition, weak C—H⋯π (ring) inter­actions, which only involve the minor disorder component. Inversion-related Cl⋯Cl halogen bonds and C—Cl⋯π (ring) contacts also help to stabilize the packing.

## Chemical context   

Schiff bases of heterocyclic amines and their complexes have attracted attention over the past decades not only due to the relatively easy synthesis, but also in view of their potential biological, pharmacological and analytical applications (Akbari *et al.*, 2017 ▸; Gurbanov *et al.*, 2018*a* ▸,*b* ▸; Hazra *et al.*, 2018 ▸; Kvyatkovskaya *et al.*, 2017 ▸; Mahmoudi *et al.*, 2016 ▸, 2017*a* ▸,*b* ▸, 2018*a* ▸,*b* ▸; Mitoraj *et al.*, 2018 ▸; Shetnev *et al.*, 2017 ▸). Non-covalent inter­actions play an important role in the stabilization of coordination or supra­molecular compounds derived from Schiff bases (Mahmudov *et al.*, 2016 ▸, 2017*a* ▸,*b* ▸; Zubkov *et al.*, 2018 ▸). Herein we report strong charge-assisted hydrogen bonds and halogen bonding in the structure of (*E*)-3-[(2,3-di­chloro­benzyl­idene)amino]-5-phenyl­thia­zolidin-2-iminium bromide.
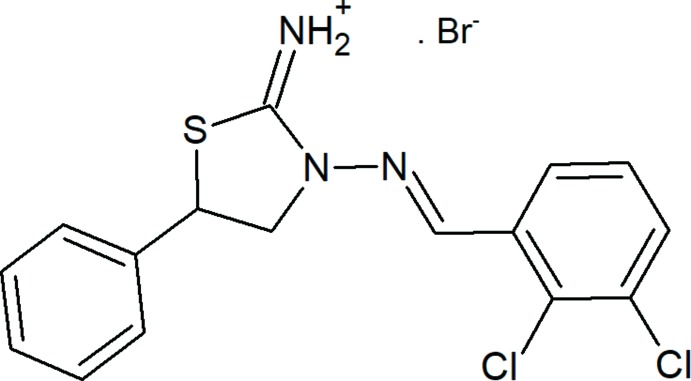


## Structural commentary   

**Figure 1:**
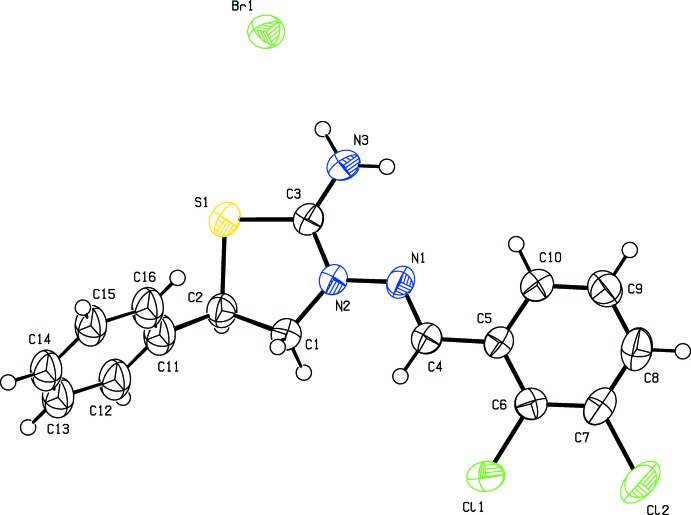
The mol­ecular structure of the title salt. Displacement ellipsoids are drawn at the 50% probability level. Hydrogen atoms are shown as spheres of arbitrary radius. The minor disorder component is omitted for clarity.

In the cation of the title salt (Fig. 1 ▸), the central thia­zolidine ring (S1/N2/C1–C3) adopts an envelope conformation with puckering parameters *Q*(2) = 0.205 (4) Å and φ(2) = 222.1 (12)°. The dihedral angle between the mean plane of the central thia­zolidine ring and the 2,3-di­chloro­benzene ring (C5–C10) is 16.0 (2)° while this plane subtends angles of 79.1 (3) and 86.7 (4)° with the major and minor components (C11–C16 and C11/C12′–C16′), respectively, of the disordered phenyl ring. The dihedral angle between the two disorder components of the ring is 7.6 (4)° and these components are oriented to the 2,3-di­chloro­benzene ring by 64.8 (3) and 72.4 (4)°, respectively. The N2—N1—C4—C5 bridge that links the thia­zolidine and 2,3-di­chloro­benzene rings has a torsion angle of 175.1 (4)°.

## Supra­molecular features and Hirshfeld surface analysis   

**Figure 2:**
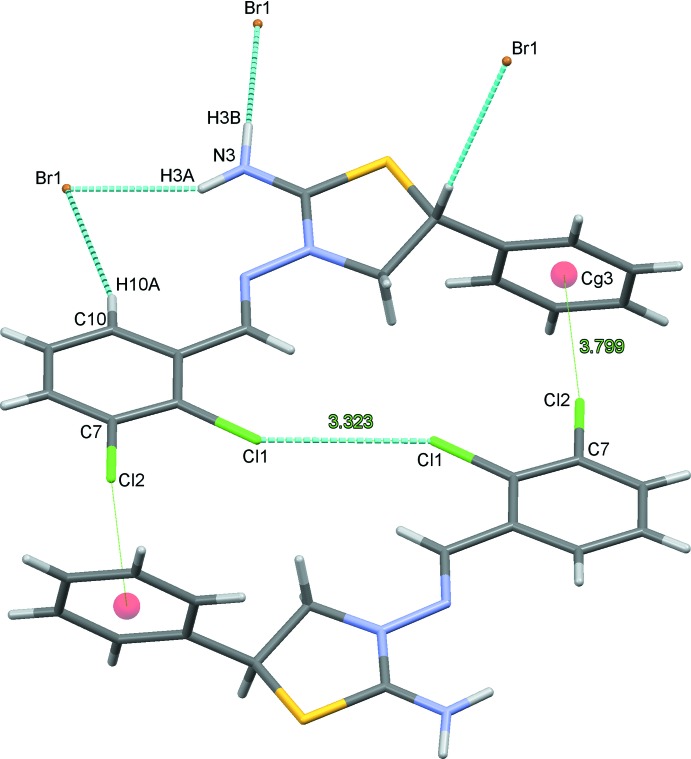
View of the full complement of contacts to an individual cation in the title salt. Only the major disorder component is shown. The symmetry-equivalent position for the cation with the label *Cg*3 is −*x* + 1, *y* − 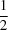, −*z* + 3/2.

**Figure 3:**
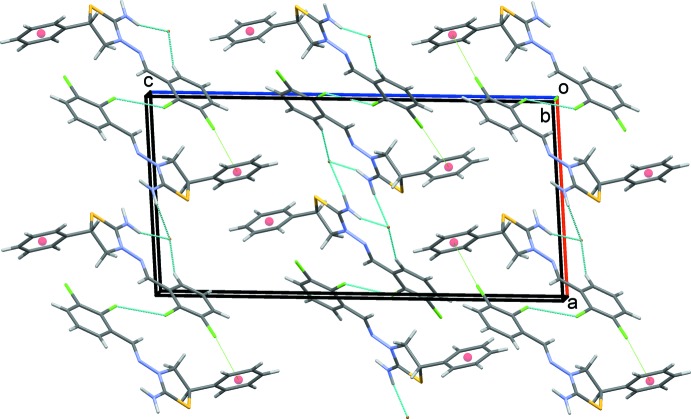
C—H⋯Br and N—H⋯Br hydrogen bonds and inversion-related Cl⋯Cl halogen bonds and C—Cl⋯π contacts of the title salt viewed along the *b* axis. Only the major disorder component is shown.

**Figure 4:**
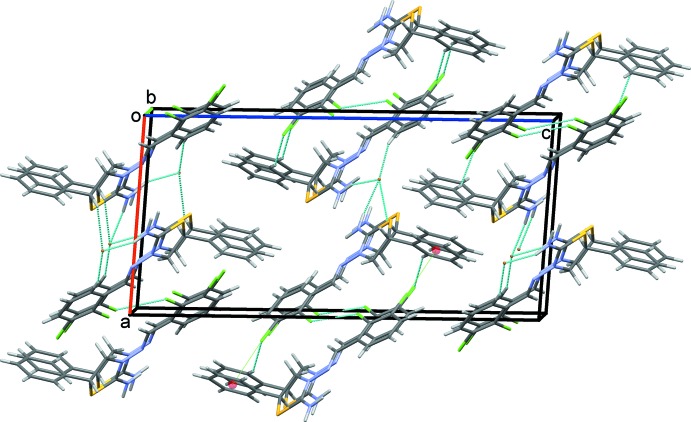
Overall packing of the title salt viewed along the *b* axis. Only the major disorder component is shown.

In the crystal, each cation forms C—H⋯Br and N—H⋯Br hydrogen bonds along with inversion-related Cl1⋯Cl1 halogen bonds and C7—Cl2⋯*Cg*3^iv^ and C7—Cl2⋯*Cg*4^iv^ contacts (Table 1 ▸; Fig. 2 ▸). Chains of cations form along the *a*-axis direction (Fig. 3 ▸). The crystal structure is further stabilized by C13′—H13*B*⋯*Cg*3^ii^ and C13′—H13*B*⋯*Cg*4^ii^ inter­actions involving the minor disorder component (Table 1 ▸). Overall, cations and anions are stacked along the *b-*axis direction (Fig. 4 ▸)

**Figure 5:**
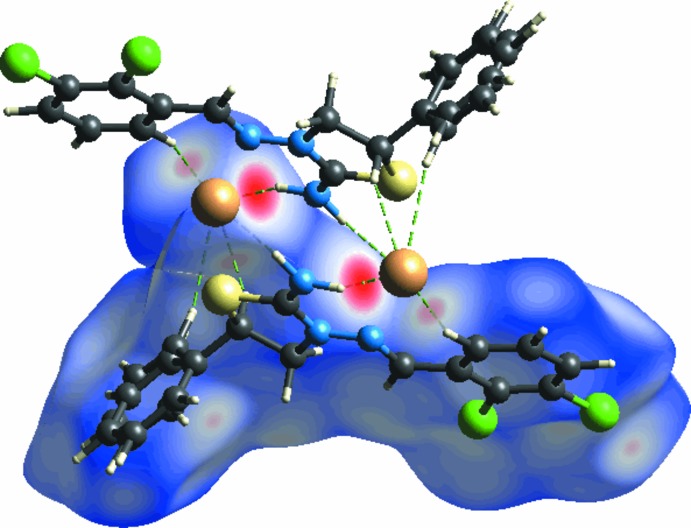
Hirshfeld surface of the title salt mapped with *d*_norm_, showing the C—H⋯Br and N—H⋯Br hydrogen bonds.

The Hirshfeld surface analysis (Spackman & Jayatilaka, 2009 ▸) of the title salt was generated by *CrystalExplorer3.1* (Wolff *et al.*, 2012 ▸), and comprised *d*_norm_ surface plots and two dimensional fingerprint plots (Spackman & McKinnon, 2002 ▸). A *d*_norm_ surface plot of the title salt is shown in Fig. 5 ▸. This plot was generated to qu­antify and visualize the inter­molecular inter­actions and to explain the observed crystal packing. The dark-red spots on the *d*_norm_ surface arise as a result of short inter­atomic contacts, while the other weaker inter­molecular inter­actions appear as light-red spots.

The *d*_norm_ surface of the title salt shows a dark-red spot at the N—H hydrogen atom and on the bromide atom, which is the result of the strong N3—H3*A*⋯Br1^i^ and N3—H3*B*⋯Br1 hydrogen bonds present in the structure (Fig. 5 ▸). Beside these two short inter­molecular contacts, the C—H⋯Br inter­action is shown as light-red spots on the *d*_norm_ surface. The short inter­atomic contacts in the title salt are given in Table 2 ▸.

**Figure 6:**
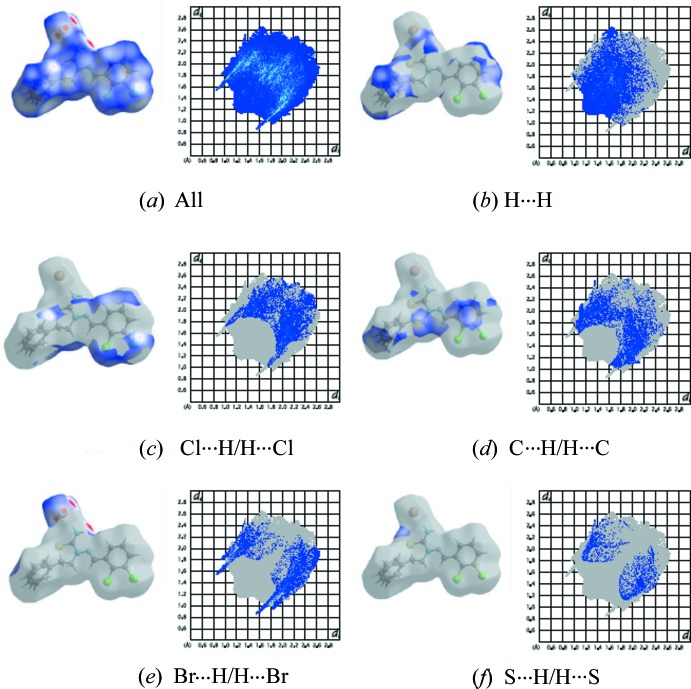
The two-dimensional fingerprint plots of the title salt, showing (*a*) all inter­actions, and delineated into (*b*) H⋯H, (*c*) Cl⋯H/H⋯Cl, (*d*) C⋯H/H⋯C, (*e*) Br⋯H/H⋯Br and (*f*) S⋯H/H⋯S inter­actions.

A qu­anti­tative analysis of the inter­molecular inter­actions can be made by studying the fingerprint plots that are shown with characteristic pseudo-symmetry wings in the *d*_e_ and *d*_i_ diagonal axes [*d*_e_ and *d*_i_ represent the distances from a point on the Hirshfeld surface to the nearest atoms outside (external) and inside (inter­nal) the surface, respectively]. These represent both the overall two-dimensional fingerprint plots and those that represent H⋯H, Cl⋯H/H⋯Cl, C⋯H/H⋯C and Br⋯H/H⋯Br contacts, respectively (Fig. 6 ▸ *b*-*e*). The most significant inter­molecular inter­actions are the H⋯H inter­action (25.4%), which appear in the central region of the fingerprint plot with *d*e = *d*i ≃ 1.2 Å (Fig. 6 ▸ *b*). The reciprocal Cl⋯H/H⋯Cl inter­actions appear as two symmetrical broad wings with *d*e + *d*i ≃ 2.8 Å and contribute 19.1% to the Hirshfeld surface (Fig. 6 ▸ *c*). The reciprocal C⋯H/H⋯C and Br⋯H/H⋯Br inter­actions with 18.2% and 16.2% contributions are present as sharp symmetrical spikes at diagonal axes *d*_e_ + *d*_i_ ≃ 2.7 and 2.4 Å, respectively (Fig. 6 ▸ *d*–*e*). The percentage contributions of other inter­molecular contacts are less than 6% in the Hirshfeld surface mapping (Table 3 ▸).

## Database survey   

A search of the Cambridge Structural Database (CSD Version 5.39, Nov 2017 plus three updates; Groom *et al.*, 2016 ▸) yielded six hits for 2-thia­zolidiniminium compounds with four of them reporting essentially the same cation: [WILBIC (Marthi *et al.*, 1994 ▸), WILBOI (Marthi *et al.*, 1994 ▸), WILBOI01 (Marthi *et al.*, 1994 ▸), YITCEJ (Martem’yanova *et al.*, 1993*a* ▸), YITCAF (Martem’yanova *et al.*, 1993*b* ▸) and YOPLUK (Marthi *et al.*, 1995 ▸)]. In all cases, the 3-N atom carries a C substituent, not N as found in the title compound. The first three crystal structures were determined for racemic (WILBIC; Marthi *et al.*, 1994 ▸) and two optically active samples (WILBOI and WILBOI01; Marthi *et al.*, 1994 ▸) of 3-(2′-chloro-2′-phenyl­eth­yl) −2-thia­zolidiniminium *p*-toluene­sulfonate. In all three structures, the most disordered fragment of these mol­ecules is the asymmetric C atom and the Cl atom attached to it. The disorder of the cation in the racemate corresponds to the presence of both enanti­omers at each site in the ratio 0.821 (3): 0.179 (3). The system of hydrogen bonds connecting two cations and two anions into 12-membered rings is identical in the racemic and in the optically active crystals. YITCEJ (Martem’yanova *et al.*, 1993*a* ▸), is a product of the inter­action of 2-amino-5-methyl­thia­zoline with methyl iodide, with alkyl­ation at the endocylic nitro­gen atom, while YITCAF (Martem’yanova *et al.*, 1993*b* ▸) is a product of the reaction of 3-nitro-5-meth­oxy-, 3-nitro-5-chloro-, and 3-bromo-5-nitro­salicyl­aldehyde with the heterocyclic base to form the salt-like complexes.

## Synthesis and crystallization   

To a solution of 1 mmol of 3-amino-5-phenyl­thia­zolidin-2-iminium bromide in 20 mL ethanol 1 mmol of 2,3-di­chloro­benzaldehyde was added and the solution refluxed for 2 h. The reaction mixture was cooled down to precipitate the product as colourless single crystals. These were collected by filtration and washed with cold acetone. The title compound was recrystallized from methanol by slow evaporation at room temperature over several days.

Yield 89%, m.p. 521 K. Analysis calculated for C_16_H_14_BrCl_2_N_3_S (*M*_r_ = 431.18): C, 44.57; H, 3.27; N, 9.75. Found: C, 44.51; H, 3.23; N, 9.72%. ^1^H NMR (300 MHz, DMSO-*d*_6_) : 4,62 (*k*, 1H, CH_2_, ^3^ *J*_H–H_ = 6.9); 4.96 (*t*, 1H, CH_2_, ^3^ *J*_H–H_ = 8.7); 5.59 (*t*, 1H, CH—Ar, ^3^ *J*_H–H_ = 7.5); 7.38–8.50 (*m*, 7H, 7Ar—H); 8.35 (*s*, 1H, CH=); 10.56 (*s*, 1H, NH=). ^13^C NMR(75 MHz, DMSO-*d*_6_): 46.62, 55.68, 127.28, 127.99, 128.48, 128.96, 129.11, 132.27, 132.41, 132.51, 133.04, 137.24, 145.89, 168.92. MS (ESI), *m*/*z*: 351.24 [C_16_H_14_Cl_2_N_3_S]^+^ and 79.88 Br^−^.

## Refinement   

Crystal data, data collection and structure refinement details are summarized in Table 4 ▸. The H atoms were positioned geometrically [N—H = 0.90 Å and C—H = 0.93–0.97 Å] and were refined using a riding model, with *U*_iso_(H) = 1.2*U*_eq_(C,N). The phenyl ring in the cation is disordered over two positions with a site occupancy ratio of 0.541 (9):0.459 (9). Using DFIX, the bond distances in the two disorder components of the phenyl ring were set to 1.40 Å. Corresponding displacement parameters were also held to be the same using EADP.

## Supplementary Material

Crystal structure: contains datablock(s) global. DOI: 10.1107/S2056989018010496/sj5561sup1.cif

Supporting information file. DOI: 10.1107/S2056989018010496/sj5561globalsup2.cml

CCDC reference: 1857411

Additional supporting information:  crystallographic information; 3D view; checkCIF report

## Figures and Tables

**Table 1:** Hydrogen-bond geometry (Å, °) *Cg*3 and *Cg*4 are the centroids of the major and minor disorder components of the C11/C12–C16 and C11/C12′–C16′ phenyl ring, respectively.

*D*—H⋯*A*	*D*—H	H⋯*A*	*D*⋯*A*	*D*—H⋯*A*
N3—H3*A*⋯Br1^i^	0.90	2.51	3.303 (4)	147
N3—H3*B*⋯Br1	0.90	2.36	3.258 (4)	175
C13′—H13*B*⋯*Cg*3^ii^	0.93	2.91	3.596 (12)	132
C13′—H13*B*⋯*Cg*4^ii^	0.93	2.99	3.746 (12)	139
C2—H2*A*⋯Br1^iii^	0.98	2.87	3.778 (5)	154
C10—H10*A*⋯Br1^i^	0.93	2.90	3.796 (5)	161
C7—Cl2⋯*Cg*3^iv^	1.73 (1)	3.80 (1)	5.525 (6)	175 (1)
C7—Cl2⋯*Cg*4^iv^	1.73 (1)	3.57 (1)	5.299 (6)	175 (1)

Symmetry codes: (i) 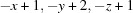; (ii) 
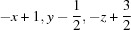
; (iii) 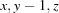; (iv) 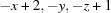.

**Table 2:** Summary of short inter­atomic contacts (Å) in the title salt Atoms marked with an asterisk (*) are from the minor component (C11/C12′–C16′) of the disordered phenyl ring of the cation.

Contact	Distance	Symmetry operation
(C6)Cl1⋯Cl1(C6)	3.323 (2)	2 − *x*, −*y*, 1 − *z*
(C16′)*H16*B*⋯H8*A*(C8)	2.56	2 − *x*, 1 − *y*, 1 − *z*
(C2)S1⋯*H14*B*(C14′)	3.05	1 − *x*, 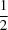 + *y*, 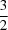 − *z*
(N3)H3*B*⋯Br1	2.36	*x*, *y*, *z*
(N3)H3*A*⋯Br1	2.51	1 − *x*, 2 − *y*, 1 − *z*
(S1)C3⋯C3(S1)	3.561 (6)	1 − *x*, 1 − *y*, 1 − *z*
(C1)H1*B*⋯Br1	3.06	1 − *x*, 1 − *y*, 1 − *z*
(C5)C10⋯*H14*B*(C14′)	2.89	*x*, 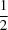 − *y*, − 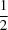 + *z*
(C14′)*H14*B*⋯S1(C2)	3.05	1 − *x*, − 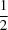 + *y*, 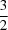 − *z*
(C14′)*H14*B*⋯C10(C5)	2.89	*x*, 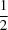 − *y*, 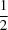 + *z*
(C2)H2*A*⋯Br1	2.87	*x*, −1 + *y*, *z*

**Table 3:** Percentage contributions of inter­atomic contacts to the Hirshfeld surface for the title salt

Contact	Percentage contribution
H⋯H	25.4
Cl⋯H/H⋯Cl	19.1
C⋯H/H⋯C	18.2
Br⋯H/H⋯Br	16.2
S⋯H/H⋯S	5.9
Cl.·C/C⋯Cl	4.4
N⋯H/H⋯N	2.7
C⋯C	1.9
Cl.·N/N⋯Cl	1.4
C.·N/N⋯C	1.3
Br.·C/C⋯Br	1.0
Cl⋯Cl	0.8
S⋯N/N⋯S	0.7
S⋯C/C⋯S	0.4
Br⋯N/N⋯Br	0.3
Br.·Cl/Cl⋯Br	0.3

**Table 4:** Experimental details

Crystal data	
Chemical formula	C_16_H_14_Cl_2_N_3_S^+^·Br^−^
*M*_r_	431.17
Crystal system, space group	Monoclinic, *P*2_1_/*c*
Temperature (K)	296
*a*, *b*, *c* (Å)	11.2586 (8), 6.8886 (5), 23.0145 (16)
β (°)	93.678 (2)
*V* (Å^3^)	1781.2 (2)
*Z*	4
Radiation type	Mo *K*α
μ (mm^−1^)	2.73
Crystal size (mm)	0.28 × 0.25 × 0.24

Data collection	
Diffractometer	Bruker APEXII CCD
Absorption correction	Multi-scan (*SADABS*; Bruker, 2007 ▸)
*T*_min_, *T*_max_	0.483, 0.546
No. of measured, independent and observed [*I* > 2σ(*I*)] reflections	20932, 3651, 2325
*R*_int_	0.085
(sin θ/λ)_max_ (Å^−1^)	0.625

Refinement	
*R*[*F* ^2^ > 2σ(*F* ^2^)], *wR*(*F* ^2^), *S*	0.051, 0.123, 1.04
No. of reflections	3651
No. of parameters	182
No. of restraints	12
H-atom treatment	H-atom parameters constrained
Δρ_max_, Δρ_min_ (e Å^−3^)	0.47, −0.61

Computer programs: *APEX2* and *SAINT* (Bruker, 2007 ▸), *SHELXS97* (Sheldrick, 2008 ▸), *SHELXL2014* (Sheldrick, 2015 ▸), *ORTEP-3 for Windows* (Farrugia, 2012 ▸), *Mercury* (Macrae *et al.*, 2008 ▸) and *PLATON* (Spek, 2003 ▸).

## supplementary crystallographic information

### Crystal data

**Table:**

C_16_H_14_Cl_2_N_3_S^+^·Br^−^	*F*(000) = 864
*M**_r_* = 431.17	*D*_x_ = 1.608 Mg m^−^^3^
Monoclinic, *P*2_1_/*c*	Mo *K*α radiation, λ = 0.71073 Å
*a* = 11.2586 (8) Å	Cell parameters from 5051 reflections
*b* = 6.8886 (5) Å	θ = 2.5–24.3°
*c* = 23.0145 (16) Å	µ = 2.73 mm^−^^1^
β = 93.678 (2)°	*T* = 296 K
*V* = 1781.2 (2) Å^3^	Block, colourless
*Z* = 4	0.28 × 0.25 × 0.24 mm

### Data collection

**Table:**

Bruker APEXII CCD diffractometer	2325 reflections with *I* > 2σ(*I*)
φ and ω scans	*R*_int_ = 0.085
Absorption correction: multi-scan (SADABS; Bruker, 2007)	θ_max_ = 26.4°, θ_min_ = 2.5°
*T*_min_ = 0.483, *T*_max_ = 0.546	*h* = −14→14
20932 measured reflections	*k* = −8→8
3651 independent reflections	*l* = −28→28

### Refinement

**Table:**

Refinement on *F*^2^	Primary atom site location: structure-invariant direct methods
Least-squares matrix: full	Secondary atom site location: difference Fourier map
*R*[*F*^2^ > 2σ(*F*^2^)] = 0.051	Hydrogen site location: mixed
*wR*(*F*^2^) = 0.123	H-atom parameters constrained
*S* = 1.04	*w* = 1/[σ^2^(*F*_o_^2^) + (0.0342*P*)^2^ + 3.7192*P*] where *P* = (*F*_o_^2^ + 2*F*_c_^2^)/3
3651 reflections	(Δ/σ)_max_ = 0.001
182 parameters	Δρ_max_ = 0.47 e Å^−^^3^
12 restraints	Δρ_min_ = −0.61 e Å^−^^3^

### Special details

**Table:**

Geometry. All esds (except the esd in the dihedral angle between two l.s. planes) are estimated using the full covariance matrix. The cell esds are taken into account individually in the estimation of esds in distances, angles and torsion angles; correlations between esds in cell parameters are only used when they are defined by crystal symmetry. An approximate (isotropic) treatment of cell esds is used for estimating esds involving l.s. planes.

### Fractional atomic coordinates and isotropic or equivalent isotropic displacement parameters (Å^2^)

**Table:**

	*x*	*y*	*z*	*U*_iso_*/*U*_eq_	Occ. (<1)
Br1	0.33815 (4)	0.95208 (8)	0.56902 (2)	0.05678 (19)	
Cl1	1.02426 (13)	0.1283 (2)	0.44079 (7)	0.0687 (4)	
Cl2	1.12966 (15)	0.2307 (2)	0.32291 (7)	0.0869 (5)	
S1	0.52713 (12)	0.4896 (2)	0.61266 (6)	0.0624 (4)	
N1	0.7591 (3)	0.5219 (5)	0.49730 (15)	0.0408 (9)	
N2	0.6952 (3)	0.4605 (6)	0.54294 (15)	0.0433 (9)	
N3	0.5794 (4)	0.7393 (6)	0.53057 (18)	0.0579 (12)	
H3A	0.624225	0.782615	0.502288	0.069*	
H3B	0.510005	0.793065	0.539518	0.069*	
C1	0.7176 (4)	0.2900 (7)	0.5795 (2)	0.0465 (11)	
H1A	0.787141	0.310790	0.605841	0.056*	
H1B	0.731818	0.177512	0.555530	0.056*	
C2	0.6072 (4)	0.2576 (7)	0.6139 (2)	0.0456 (11)	
H2A	0.556596	0.159930	0.593684	0.055*	
C3	0.6050 (4)	0.5758 (7)	0.5566 (2)	0.0436 (11)	
C4	0.8437 (4)	0.4154 (6)	0.48214 (18)	0.0380 (10)	
H4A	0.865728	0.304542	0.503231	0.046*	
C5	0.9060 (4)	0.4708 (6)	0.43080 (18)	0.0368 (10)	
C6	0.9857 (4)	0.3433 (6)	0.40648 (19)	0.0414 (10)	
C7	1.0339 (4)	0.3904 (8)	0.3543 (2)	0.0514 (13)	
C8	1.0074 (4)	0.5637 (9)	0.3272 (2)	0.0608 (14)	
H8A	1.039350	0.593679	0.292068	0.073*	
C9	0.9330 (5)	0.6929 (8)	0.3525 (2)	0.0586 (14)	
H9A	0.916921	0.812359	0.334891	0.070*	
C10	0.8825 (4)	0.6470 (7)	0.4033 (2)	0.0487 (12)	
H10A	0.831686	0.735272	0.419627	0.058*	
C11	0.6345 (4)	0.1918 (6)	0.67564 (14)	0.0609 (8)	
C12	0.6080 (6)	0.0096 (6)	0.6973 (3)	0.0609 (8)	0.541 (9)
H12A	0.571907	−0.083422	0.672760	0.073*	0.541 (9)
C13	0.6355 (8)	−0.0337 (10)	0.7556 (3)	0.0609 (8)	0.541 (9)
H13A	0.617773	−0.155583	0.770084	0.073*	0.541 (9)
C14	0.6895 (7)	0.1053 (15)	0.79226 (18)	0.0609 (8)	0.541 (9)
H14A	0.707872	0.076397	0.831271	0.073*	0.541 (9)
C15	0.7160 (6)	0.2875 (13)	0.7706 (2)	0.0609 (8)	0.541 (9)
H15A	0.752105	0.380540	0.795135	0.073*	0.541 (9)
C16	0.6885 (6)	0.3308 (7)	0.7123 (2)	0.0609 (8)	0.541 (9)
H16A	0.706240	0.452706	0.697811	0.073*	0.541 (9)
C12'	0.5874 (10)	0.0071 (10)	0.6850 (4)	0.0609 (8)	0.459 (9)
H12B	0.540937	−0.050445	0.654817	0.073*	0.459 (9)
C13'	0.6064 (10)	−0.0955 (17)	0.7373 (4)	0.0609 (8)	0.459 (9)
H13B	0.575083	−0.219058	0.741912	0.073*	0.459 (9)
C14'	0.6746 (10)	−0.0036 (19)	0.7822 (5)	0.0609 (8)	0.459 (9)
H14B	0.690965	−0.069494	0.817074	0.073*	0.459 (9)
C15'	0.7188 (10)	0.1846 (18)	0.7762 (4)	0.0609 (8)	0.459 (9)
H15B	0.758883	0.247056	0.807429	0.073*	0.459 (9)
C16'	0.7016 (9)	0.2771 (16)	0.7221 (3)	0.0609 (8)	0.459 (9)
H16B	0.735675	0.398420	0.716985	0.073*	0.459 (9)

### Atomic displacement parameters (Å^2^)

**Table:**

	*U*^11^	*U*^22^	*U*^33^	*U*^12^	*U*^13^	*U*^23^
Br1	0.0494 (3)	0.0517 (3)	0.0702 (4)	0.0042 (3)	0.0114 (2)	0.0059 (3)
Cl1	0.0778 (9)	0.0476 (8)	0.0840 (10)	0.0186 (7)	0.0307 (8)	0.0078 (7)
Cl2	0.0899 (11)	0.0856 (11)	0.0910 (12)	0.0098 (9)	0.0511 (9)	−0.0178 (9)
S1	0.0564 (8)	0.0674 (9)	0.0669 (9)	0.0151 (7)	0.0308 (7)	0.0139 (7)
N1	0.041 (2)	0.044 (2)	0.039 (2)	−0.0015 (17)	0.0120 (16)	−0.0025 (17)
N2	0.045 (2)	0.046 (2)	0.041 (2)	0.0077 (18)	0.0128 (17)	0.0048 (18)
N3	0.052 (2)	0.057 (3)	0.067 (3)	0.019 (2)	0.025 (2)	0.011 (2)
C1	0.049 (3)	0.049 (3)	0.042 (3)	0.007 (2)	0.009 (2)	0.004 (2)
C2	0.043 (3)	0.051 (3)	0.044 (3)	−0.001 (2)	0.008 (2)	0.003 (2)
C3	0.042 (2)	0.045 (3)	0.044 (3)	0.002 (2)	0.009 (2)	0.001 (2)
C4	0.038 (2)	0.039 (3)	0.037 (2)	−0.0026 (19)	0.0010 (19)	−0.0036 (19)
C5	0.033 (2)	0.040 (2)	0.037 (2)	−0.0042 (19)	0.0035 (18)	−0.005 (2)
C6	0.039 (2)	0.039 (3)	0.046 (3)	−0.004 (2)	0.002 (2)	−0.002 (2)
C7	0.048 (3)	0.059 (3)	0.049 (3)	−0.004 (2)	0.015 (2)	−0.012 (3)
C8	0.051 (3)	0.084 (4)	0.048 (3)	−0.004 (3)	0.012 (2)	0.004 (3)
C9	0.055 (3)	0.061 (3)	0.060 (3)	0.005 (3)	0.007 (3)	0.015 (3)
C10	0.047 (3)	0.050 (3)	0.049 (3)	0.006 (2)	0.008 (2)	0.000 (2)
C11	0.0495 (16)	0.090 (2)	0.0436 (16)	0.0106 (16)	0.0043 (12)	0.0150 (15)
C12	0.0495 (16)	0.090 (2)	0.0436 (16)	0.0106 (16)	0.0043 (12)	0.0150 (15)
C13	0.0495 (16)	0.090 (2)	0.0436 (16)	0.0106 (16)	0.0043 (12)	0.0150 (15)
C14	0.0495 (16)	0.090 (2)	0.0436 (16)	0.0106 (16)	0.0043 (12)	0.0150 (15)
C15	0.0495 (16)	0.090 (2)	0.0436 (16)	0.0106 (16)	0.0043 (12)	0.0150 (15)
C16	0.0495 (16)	0.090 (2)	0.0436 (16)	0.0106 (16)	0.0043 (12)	0.0150 (15)
C12'	0.0495 (16)	0.090 (2)	0.0436 (16)	0.0106 (16)	0.0043 (12)	0.0150 (15)
C13'	0.0495 (16)	0.090 (2)	0.0436 (16)	0.0106 (16)	0.0043 (12)	0.0150 (15)
C14'	0.0495 (16)	0.090 (2)	0.0436 (16)	0.0106 (16)	0.0043 (12)	0.0150 (15)
C15'	0.0495 (16)	0.090 (2)	0.0436 (16)	0.0106 (16)	0.0043 (12)	0.0150 (15)
C16'	0.0495 (16)	0.090 (2)	0.0436 (16)	0.0106 (16)	0.0043 (12)	0.0150 (15)

### Geometric parameters (Å, º)

**Table:**

Cl1—C6	1.720 (5)	C9—H9A	0.9300
Cl2—C7	1.730 (5)	C10—H10A	0.9300
S1—C3	1.712 (5)	C11—C12	1.3900
S1—C2	1.834 (5)	C11—C16	1.3900
N1—C4	1.269 (5)	C11—C16'	1.399 (2)
N1—N2	1.377 (5)	C11—C12'	1.400 (2)
N2—C3	1.342 (5)	C12—C13	1.3900
N2—C1	1.457 (6)	C12—H12A	0.9300
N3—C3	1.299 (6)	C13—C14	1.3900
N3—H3A	0.9000	C13—H13A	0.9300
N3—H3B	0.9001	C14—C15	1.3900
C1—C2	1.533 (6)	C14—H14A	0.9300
C1—H1A	0.9700	C15—C16	1.3900
C1—H1B	0.9700	C15—H15A	0.9300
C2—C11	1.503 (6)	C16—H16A	0.9300
C2—H2A	0.9800	C12'—C13'	1.400 (2)
C4—C5	1.463 (6)	C12'—H12B	0.9300
C4—H4A	0.9300	C13'—C14'	1.400 (2)
C5—C10	1.386 (6)	C13'—H13B	0.9300
C5—C6	1.398 (6)	C14'—C15'	1.399 (2)
C6—C7	1.388 (6)	C14'—H14B	0.9300
C7—C8	1.370 (7)	C15'—C16'	1.400 (2)
C8—C9	1.376 (7)	C15'—H15B	0.9300
C8—H8A	0.9300	C16'—H16B	0.9300
C9—C10	1.371 (7)		
			
C3—S1—C2	92.3 (2)	C8—C9—H9A	119.7
C4—N1—N2	118.1 (4)	C9—C10—C5	120.9 (5)
C3—N2—N1	115.9 (4)	C9—C10—H10A	119.5
C3—N2—C1	116.6 (4)	C5—C10—H10A	119.5
N1—N2—C1	127.4 (3)	C12—C11—C16	120.0
C3—N3—H3A	120.2	C16'—C11—C12'	117.1 (6)
C3—N3—H3B	114.9	C12—C11—C2	125.1 (4)
H3A—N3—H3B	124.4	C16—C11—C2	114.9 (4)
N2—C1—C2	107.4 (4)	C16'—C11—C2	131.6 (6)
N2—C1—H1A	110.2	C12'—C11—C2	111.2 (5)
C2—C1—H1A	110.2	C13—C12—C11	120.0
N2—C1—H1B	110.2	C13—C12—H12A	120.0
C2—C1—H1B	110.2	C11—C12—H12A	120.0
H1A—C1—H1B	108.5	C12—C13—C14	120.0
C11—C2—C1	114.2 (4)	C12—C13—H13A	120.0
C11—C2—S1	110.4 (3)	C14—C13—H13A	120.0
C1—C2—S1	106.2 (3)	C13—C14—C15	120.0
C11—C2—H2A	108.7	C13—C14—H14A	120.0
C1—C2—H2A	108.7	C15—C14—H14A	120.0
S1—C2—H2A	108.7	C16—C15—C14	120.0
N3—C3—N2	123.6 (4)	C16—C15—H15A	120.0
N3—C3—S1	122.6 (3)	C14—C15—H15A	120.0
N2—C3—S1	113.8 (3)	C15—C16—C11	120.0
N1—C4—C5	118.6 (4)	C15—C16—H16A	120.0
N1—C4—H4A	120.7	C11—C16—H16A	120.0
C5—C4—H4A	120.7	C11—C12'—C13'	123.5 (9)
C10—C5—C6	118.5 (4)	C11—C12'—H12B	118.3
C10—C5—C4	120.6 (4)	C13'—C12'—H12B	118.3
C6—C5—C4	120.8 (4)	C14'—C13'—C12'	117.0 (10)
C7—C6—C5	119.7 (4)	C14'—C13'—H13B	121.5
C7—C6—Cl1	119.8 (4)	C12'—C13'—H13B	121.5
C5—C6—Cl1	120.4 (3)	C15'—C14'—C13'	121.8 (10)
C8—C7—C6	120.7 (4)	C15'—C14'—H14B	119.1
C8—C7—Cl2	119.2 (4)	C13'—C14'—H14B	119.1
C6—C7—Cl2	120.1 (4)	C14'—C15'—C16'	118.7 (10)
C7—C8—C9	119.5 (5)	C14'—C15'—H15B	120.6
C7—C8—H8A	120.2	C16'—C15'—H15B	120.6
C9—C8—H8A	120.2	C11—C16'—C15'	121.7 (8)
C10—C9—C8	120.6 (5)	C11—C16'—H16B	119.2
C10—C9—H9A	119.7	C15'—C16'—H16B	119.2
			
C4—N1—N2—C3	−178.7 (4)	C8—C9—C10—C5	0.7 (8)
C4—N1—N2—C1	4.3 (6)	C6—C5—C10—C9	2.1 (7)
C3—N2—C1—C2	16.1 (6)	C4—C5—C10—C9	−174.5 (4)
N1—N2—C1—C2	−166.9 (4)	C1—C2—C11—C12	−112.2 (5)
N2—C1—C2—C11	−141.6 (4)	S1—C2—C11—C12	128.3 (4)
N2—C1—C2—S1	−19.8 (4)	C1—C2—C11—C16	69.0 (5)
C3—S1—C2—C11	140.2 (3)	S1—C2—C11—C16	−50.4 (4)
C3—S1—C2—C1	15.9 (4)	C1—C2—C11—C16'	58.9 (10)
N1—N2—C3—N3	−2.2 (7)	S1—C2—C11—C16'	−60.6 (9)
C1—N2—C3—N3	175.2 (5)	C1—C2—C11—C12'	−117.4 (7)
N1—N2—C3—S1	178.8 (3)	S1—C2—C11—C12'	123.1 (7)
C1—N2—C3—S1	−3.8 (5)	C16—C11—C12—C13	0.0
C2—S1—C3—N3	173.2 (4)	C2—C11—C12—C13	−178.7 (5)
C2—S1—C3—N2	−7.8 (4)	C11—C12—C13—C14	0.0
N2—N1—C4—C5	175.1 (4)	C12—C13—C14—C15	0.0
N1—C4—C5—C10	7.4 (6)	C13—C14—C15—C16	0.0
N1—C4—C5—C6	−169.2 (4)	C14—C15—C16—C11	0.0
C10—C5—C6—C7	−3.5 (6)	C12—C11—C16—C15	0.0
C4—C5—C6—C7	173.1 (4)	C2—C11—C16—C15	178.9 (4)
C10—C5—C6—Cl1	176.6 (3)	C16'—C11—C12'—C13'	−1.5 (15)
C4—C5—C6—Cl1	−6.8 (6)	C2—C11—C12'—C13'	175.4 (9)
C5—C6—C7—C8	2.2 (7)	C11—C12'—C13'—C14'	1.2 (16)
Cl1—C6—C7—C8	−177.9 (4)	C12'—C13'—C14'—C15'	2.0 (16)
C5—C6—C7—Cl2	−178.4 (3)	C13'—C14'—C15'—C16'	−4.9 (16)
Cl1—C6—C7—Cl2	1.6 (6)	C12'—C11—C16'—C15'	−1.5 (15)
C6—C7—C8—C9	0.6 (8)	C2—C11—C16'—C15'	−177.6 (7)
Cl2—C7—C8—C9	−178.8 (4)	C14'—C15'—C16'—C11	4.6 (16)
C7—C8—C9—C10	−2.1 (8)		

### Hydrogen-bond geometry (Å, º)

Cg3 and Cg4 are the centroids of the major (C11-C16) and minor (C11/C12'–C16') disorder components, respectively, of the phenyl ring.

**Table:**

*D*—H···*A*	*D*—H	H···*A*	*D*···*A*	*D*—H···*A*
N3—H3*A*···Br1^i^	0.90	2.51	3.303 (4)	147
N3—H3*B*···Br1	0.90	2.36	3.258 (4)	175
C13′—H13*B*···*Cg*3^ii^	0.93	2.91	3.596 (12)	132
C13′—H13*B*···*Cg*4^ii^	0.93	2.99	3.746 (12)	139
C2—H2*A*···Br1^iii^	0.98	2.87	3.778 (5)	154
C10—H10*A*···Br1^i^	0.93	2.90	3.796 (5)	161
C7—Cl2···*Cg*3^iv^	1.73 (1)	3.80 (1)	5.525 (6)	175 (1)
C7—Cl2···*Cg*4^iv^	1.73 (1)	3.57 (1)	5.299 (6)	175 (1)

Symmetry codes: (i) −*x*+1, −*y*+2, −*z*+1; (ii) −*x*+1, *y*−1/2, −*z*+3/2; (iii) *x*, *y*−1, *z*; (iv) −*x*+2, −*y*, −*z*+1.
